# Insulin Resistance, Temperament and Personality Traits Are Associated with Anhedonia in a Transdiagnostic Sample

**DOI:** 10.3390/brainsci14090890

**Published:** 2024-08-31

**Authors:** Marcin Siwek, Adrian A. Chrobak, Zbigniew Sołtys, Dominika Dudek, Anna J. Krupa

**Affiliations:** 1 Department of Affective Disorders, Jagiellonian University Medical College, Kopernika St. 21a, 31-501 Krakow, Poland; marcin.siwek@uj.edu.pl; 2 Department of Adult Psychiatry, Jagiellonian University Medical College, Kopernika St. 21a, 31-501 Krakow, Poland; adrian.chrobak@uj.edu.pl (A.A.C.); dominika.dudek@poczta.fm (D.D.); 3 Laboratory of Experimental Neuropathology, Institute of Zoology and Biomedical Research, Jagiellonian University, Gronostajowa 9, 30-387 Krakow, Poland; soltysza@yahoo.com

**Keywords:** anhedonia, insulin resistance, major depression, affective temperament, personality, schizotypy

## Abstract

Anhedonia constitutes a core symptom of major depressive disorder (MDD) mediating the ultimate goal of MDD treatment: functional remission. Anhedonia is also present in other clinical populations, including patients with chronic pain. Recent data links anhedonia to insulin resistance (IR). Some researchers have underlined a different dimension of anhedonia as a temperament/personality trait. The objective of this post-hoc analysis was to explore the links between anhedonia (main outcome) and (1) IR, (2) temperamental, personality, and schizotypy traits (exposures). The study population included patients with MDD, fibromyalgia, and healthy controls. Participants were split into groups: (1) insulin resistant (IR[+] *n* = 69, HOMA-IR ≥ 2.1) and (2) insulin sensitive (IR[−] *n* = 69, HOMA-IR < 2.1). Anhedonia was significantly higher in the IR[+] group than the IR[−] group. IR was a predictor of higher anhedonia levels. IR[+] vs. IR[−] participants showed higher levels of anxiety and lower levels of hyperthymic affective temperaments, as well as conscientiousness and emotional stability personality traits. Depressive, irritable, and anxious temperaments, cognitive disorganization, and introvertive anhedonia positively predicted anhedonia, while hyperthymic temperament, conscientiousness, extraversion, and emotional stability traits negatively predicted anhedonia. IR partially mediated the relationship between depressive temperament and anhedonia. In sum, IR, affective temperaments, and personality traits are predictors of anhedonia.

## 1. Introduction

Anhedonia is a multidimensional phenomenon described as a loss/diminished capacity for and/or interest in anticipating, pursuing, and experiencing pleasure, and it constitutes the core symptom of major depressive disorder (MDD) [1]. Indeed, according to the Diagnostic Statistical Manual 5th version (DSM-5), major symptoms mandating a diagnosis of MDD if they cause significant distress or functional impairment are: depressed mood and/or loss of interest or pleasure which lasts for at least 2 weeks, accompanied by significant increase or decrease in weight/appetite, insomnia/hypersomnia, psychomotor agitation/retardation, fatigue/loss of energy, feelings of worthlessness or excessive guilt, compromised ability to think, concentrate, and make decisions, and thoughts of deaths/suicidal ideation or tendencies [2]. Anhedonia is also noted in other groups of patients, i.e., those with chronic pain, substance use disorders, schizophrenia, and Parkinson’s disease. Notably, in these conditions alone (without comorbid MDD), anhedonia is less severe than in current MDD [3]. This is an important distinction because it should be noted that comorbid MDD affects around 50% of all of these patient populations [4,5,6,7]. Indeed, in a meta-analysis including a mixed group of chronic pain patients, Garland et al. [8] found that 25% of them reported significant severity of anhedonia, and the variance of anhedonia scores was only partially explained by depression, while opioid misuse was a significant predictor of anhedonia. In our previous work we noted that anhedonia was significantly more common and more severe in patients with fibromyalgia (FM) compared to healthy participants. Furthermore, we noted that in FM subjects, higher anhedonia was linked to lack of response to treatment with serotonin and noradrenaline reuptake inhibitors (SNRI) [9]. While the data on anhedonia in chronic pain is limited, it seems that in chronic pain patients, anhedonia does not differ qualitatively from that observed in MDD [3]. Another perspective on hedonic capacity was proposed, which describes it as a relatively stable temperamental or personality trait. Meehl [10] as well as Mason and Claridge [11] included anhedonia as one component of schizotypy. In congruence with the understanding of affective temperament types as existing on a continuum ranging from subclinical presentations to serious psychopathology [12], Walsh et al. [13] reported that hyperthymic features of affective temperament were linked to positive affect and positive evaluation of the subject’s situation. Conversely, cyclothymic and irritable features were positively related to negative affect as well as the subject’s assessment of their current situation as stressful and negative, and dysthymic features were related to negative affect in a non-clinical sample of participants deemed at-risk for bipolar spectrum psychopathology after psychometric assessments. Moreover, it was recently shown in a transdiagnostic community sample that it is personality characteristics and not clinical symptoms of depression or anxiety that are associated with anhedonia levels [14]. 

Lately, evidence is emerging to support the links between insulin resistance (IR) and a specific “metabolic” MDD subtype which is distinguished from other MDD presentations by pathophysiological changes, clinical presentation, and varying response to treatment [15]. Data from animal and human studies indicate that insulin has a direct impact on serotoninergic and dopaminergic transmission, which are of clue importance to mood and hedonic tone, and that this relationship becomes disturbed in subjects with IR [16,17]. Furthermore, in an adolescent sample, Singh et al. observed that MDD subjects with IR presented higher severity of anhedonia than insulin sensitive ones [18]. Brouwer et al. found that MDD individuals with comorbid type 2 diabetes (T2D) with higher IR had significantly more pronounced anhedonia, irritability, fatigue, and hypersomnia compared to those with lower IR [19]. What is more, Rashidian et al. noted that IR is associated with slower improvement in anhedonia, worse cognitive and general functioning, and non-response to vortioxetine [20,21]. Recently, we noted that in MDD subjects treated with SNRI, IR is weakly but significantly correlated with anhedonia, psychomotor symptoms, appetite/weight, levels of energy, depressed mood, suicidal ideation, self-criticism, and sleep disturbance [22].

Unfortunately, the drugs most often prescribed in MDD and chronic pain treatment, selective serotonin reuptake inhibitors (SSRIs) and SNRIs [23,24], might not only have limited effectiveness in restoration of positive affect, but they might also induce numbness, indifference, and restrict the scope of experienced affective states and the ability to react to them [25,26,27]. This is a critical problem in MDD treatment, because as a reduction in excess negative affect leads to symptomatic response and remission, improvements in positive affect mediate functional remission, which is the main goal of the maintenance phase of MDD treatment [25,26,27,28,29]. Given the mechanism of SSRIs and SNRIs, which indiscriminately enhance serotoninergic signaling, they might restrict the dopaminergic and noradrenergic transmission in the prefrontal cortex (PFC) via 5HT2, 5HT3, and 5HT7 stimulation and hence might dampen positive affect [25,30,31,32]. However, even antidepressants with more complex receptor profiles which modulate dopaminergic, noradrenergic, or glutamatergic neurotransmission in PFC, such as vortioxetine [20,21,25], trazodone [33,34,35], agomelatine [36,37], or ketamine [38,39], do not reinstate the optimal hedonic tone in all patients, suggesting some other pathophysiology is also of importance in this regard. Therefore, it is critical that the research focuses on a broader understanding of anhedonia, its biological underpinnings, and the potential links between its psychopathological and constitutional dimensions.

The aim of this work was to transdiagnostically explore the links between anhedonia and (1) IR, (2) psychological variables of temperament, personality, and schizotypy.

## 2. Materials and Methods

### 2.1. Recruitment and Participants

In this work we pooled data on IR, constitutional psychological variables, and anhedonia from different stages of a larger cross-sectional study which aimed to verify the associations between metabolic, chronobiological, and psychopathological variables and response to antidepressants. The main outcome of this analysis was to identify the links between anhedonia and two analyzed types of exposure: (1) IR, and (2) temperamental, personality, and schizotypal features. The population for this post-hoc analysis included data of patients with MDD, patients with FM, and healthy controls (HC). For this analysis, this population was split into two transdiagnostic groups of (1) insulin resistant participants (IR[+]) and (2) a control group of insulin sensitive individuals (IR[−]). 

For the prior study from which the data were pooled, the subjects were recruited from the psychiatry as well as rheumatology and immunology departments. The inclusion criteria for the MDD group were (1) diagnosis of MDD according to the International Statistical Classification of Diseases-10th revision and DSM-5, (2) age 18–65, and (3) history of treatment with SNRI duloxetine (60–120 mg/d) or venlafaxine (150–225 mg/d) of ≥8 weeks duration. The inclusion criteria for the FM group were (1) rheumatologist-confirmed diagnosis of fibromyalgia according to the 2016 American College of Rheumatology criteria [40], (2) age 18–65 years old, and (3) history of SNRI pharmacotherapy with duloxetine (60–120 mg/d), venlafaxine (150–225 mg/d), or milnacipran (100–200 mg/d).

In both the MDD and FM patient groups the exclusion criteria were largely similar, as follows: (1) diagnosis of diabetes mellitus, (2) presence of any other severe, acute, or chronic neurological or musculoskeletal pain or other somatic disorders, (3) substance use disorder (SUD) (other than nicotine), (4) history of psychotic symptoms, (5) diagnosis of severe mental illness (schizophrenia spectrum or bipolar disorder) or severe personality disorder as classified by Tyrer et al. [41], and (6) no history of SNRI treatment, history of taking subtherapeutic SNRI doses, or history of taking an SNRI for <8 weeks. To clarify, we did not include FM patients in the MDD group, but in the FM group we allowed for the participation of individuals with comorbid depression, anxiety, and mild/moderate personality disorders because of a rather high prevalence of these psychiatric disorders in FM, which have a lifetime occurrence of 9–27%, 5–76%, and 19.3% in this population [42].

Furthermore, a group of HC was recruited. The inclusion criterion was that the participants were between 18 and 65 years old. The exclusion criteria for this group were: (1) severe, acute, or chronic psychiatric disorders, (2) severe, acute, or chronic somatic disorders, and (3) SUD (other than nicotine). 

Each participant was assessed by a physician who collected the demographic and clinical data. We allowed for the inclusion of subjects with comorbidities (asthma, allergies, dermatoses, thyroid insufficiency, hyperlipidemia, and hypertension) on condition that these were appropriately treated and well controlled (to assure this, subjects provided certificates from their attending physicians or laboratory test results).

All participants provided an informed written consent. Both stages of the study were approved by the local Bioethical Committee (No. 1072.6120.172.2021; No. 1072.6120.276.2022).

### 2.2. Laboratory Tests

Venous blood samples were collected from participants after at least 12 h of fasting. The assessments included serum levels of glucose and insulin; the tests were executed by a certified diagnostic laboratory. Based on the obtained data, the HOMA-IR (homeostasis model assessment of insulin resistance) was calculated to measure the level of IR [43]). It has been reported that HOMA-IR levels are different among various populations [44]; hence, to define IR, we used available data for the Polish population, which identified values of >2.1 for HOMA-IR as above-threshold (based on the cut-off value >75th percentile) [45,46]. Participants were divided into two groups of either insulin sensitive (IR[−]) or insulin resistant (IR[+]) depending on their HOMA-IR (IS if HOMA-IR ≤ 2.1, or IR if HOMA-IR > 2.1). 

### 2.3. Evaluation of Psychological Variables

All subjects completed self-report tools to assess:

(1) Anhedonia: the Snaith–Hamilton Pleasure Scale (SHAPS) was used, which consists of 14 statements about everyday pleasurable situations covering the domains of social interaction, foods/drinks, sensory experiences, and achievement/pastimes. Subjects responded by choosing (strongly agree, agree, disagree, or strongly disagree) according to how they had felt in the past few days. SHAPS is assessed as a 2-point scoring scale (strongly agree/agree = 0; disagree/strongly disagree = 1), with a total possible score of 14 and the cut-off for clinical level anhedonia being >2 [47,48,49].
(2) Affective temperaments: the Temperament Scale of Memphis, Pisa, and San Diego, self-administered version (TEMPS-A) was used, which assesses depressive, cyclothymic, hyperthymic, irritable, and anxious temperamental features [12,50,51].
(3) Personality traits: the Ten-Item Personality Inventory (TIPI) was used, which is a 10-item inventory measuring the “Big Five” personality dimensions, namely, extraversion/introversion, agreeableness, conscientiousness, emotional stability/neuroticism, and openness to experience [52,53].
(4) Schizotypy: the Oxford–Liverpool Inventory of Feelings and Experiences (O-LIFE) was used, which was created to assess schizotypy in healthy individuals [11,54,55].

### 2.4. Study Sample

The analyzed sample consisted of 51 MDD, 57 FM, and 30 HC subjects. The MDD, FM, and HC groups and the recruitment process have already been described in our previous works [9,22,56,57,58]. Moreover, only 51 (of 67 reported for previous work) subjects with MDD and 57 FM patients (of 62 reported in earlier articles) were included for this analysis due to missing data. All 30 HC were included, as all the data for this group were available. Nonetheless, in this work we reanalyzed the data from a different perspective, and split individuals into IR[−] and IR[+] subgroups.

### 2.5. Statistical Analysis

Basic statistical analyses were performed with the use of R software (version 4.4.1) [59] and functions from the ‘rstatix’ package. For quantitative data, a *t*-test was applied, with corrections for nonhomogeneous variance in the cases of significant Levene’s test. For qualitative data, a Chi-squared test was used. In addition, ANCOVA was used to test for possible confounding effects of physiological variables on the significance of differences in psychological parameters. Mediation analysis was performed with the ‘mediation’ package [60]. For visualization of the results, the ‘ggplot’ package was used [61].

## 3. Results

### 3.1. General and Metabolic Group Characteristics

The analyzed sample consisted of 69 IR[−] and 69 IR[+] subjects. Both groups were comparable regarding mean age, sex, and proportions of subjects with hyperlipidemia, hypertension, and hypothyroidism. The proportion of smokers vs. non-smokers was higher in IR[+] vs. IR[−]. IR[+] subjects were characterized by higher mean BMI, fasting glucose, fasting insulin, and HOMA-IR vs. IR[−]. Additionally, subjects from different diagnostic groups (MDD/FM/HC) showed significant differences in the proportion of IR[+] vs. IR[−] (Table 1). 

### 3.2. Anhedonia, Temperament and Personality

**Figure 1:**
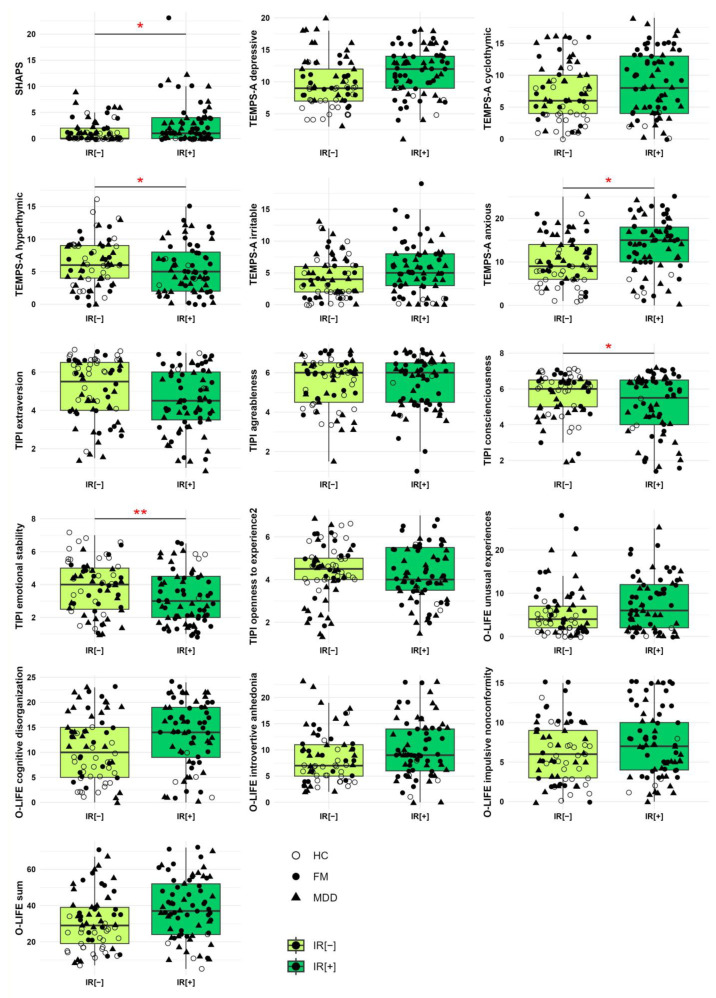
Box-plot comparisons of anhedonia and features of temperament, traits of personality, and schizotypy in the studied groups. Horizontal lines in the boxes visualize medians, boxes represent interquartile range (IQR), and whiskers show 1.5 IQR. HC, healthy controls; IR[−], insulin sensitive subjects; IR[+], insulin resistant subjects; FM, fibromyalgia subjects; MDD, major depression subjects. * *p* < 0.05, ** *p* < 0.01; p.corr, *p* value adjusted for BMI and hyperlipidemia (ANCOVA).

The IR[+] group presented significantly higher levels of anhedonia than the IR[−] group (p.corr = 0.033; η^2^ = 0.57). IR[+] participants showed significantly higher levels of anxious temperament (p.corr = 0.02; η^2^ = 0.38) and lower levels of hyperthymic temperament (p.corr = 0.012, η^2^ = 0.042) vs. IR[−]. Moreover, IR[−] participants had significantly higher levels of conscientiousness (p.corr = 0.049; g = 0.31) and emotional stability (lower neuroticism) (p.corr = 0.005; η^2^ = 0.057) compared to IR[+] participants (Table 2, Figure 1).

### 3.3. Associations between Anhedonia, IR, Temperaments and Personality

Regression analyses indicated that IR (*p* = 0.032; R^2^ = 0.026) (but not BMI) was a positive predictor of anhedonia. What is more, features of depressive (*p* = 0.001; R^2^ = 0.068) and anxious (*p* < 0.001; R^2^ = 0.079) temperaments and schizotypy dimensions of cognitive disorganization (*p* < 0.001; R^2^ = 0.104) and introvertive anhedonia (*p* < 0.001; R^2^ = 0.148), as well as overall schizotypy (*p* = 0.001; R^2^ = 0.067), were positive predictors of anhedonia. On the other hand, features of hyperthymic temperament (*p* < 0.001; R^2^ = 0.109) and certain personality traits, i.e., extraversion (*p* < 0.001; R^2^ = 0.226), conscientiousness (*p* = 0.014; R^2^ = 0.037), and emotional stability (*p* < 0.001; R^2^ = 0.096), were negative predictors of anhedonia (Table 3).

The analyses of associations between metabolic and psychological variables and level of anhedonia showed that IR, as measured by HOMA-IR, partially mediated the variability in the relationships between depressive temperament and anhedonia (Appendix A Table A1).

## 4. Discussion

This analysis was carried out to explore associations between anhedonia and IR, as well as levels of temperamental, personality, and schizotypy traits. Our work is innovative in that it bridges three perspectives on anhedonia as a state symptom of MDD (or other pathologies, e.g., chronic pain disorders), a trait related to constitutional features of temperament or personality, and a psychological manifestation of dysregulation of insulin signaling. Additionally, it implies that the associations between the state and trait dimensions of hedonic tone might be partially mediated via IR. 

Firstly, we found that anhedonia was significantly higher in IR[+] than IR[−] subjects and that IR (but not BMI) was a positive predictor of higher anhedonia levels (Table 2, Figure 1). At first glance this is in contrast with the previous data, which indicated that in overweight/obese subjects the functioning of reward circuits is impaired [62] and that longitudinal shifts from normal to overweight or overweight to chronically obese are associated with higher anhedonia levels [63]. Yet, it seems that the relationship between anhedonia, BMI, and insulin are not only complex, but most likely also dynamic. Indeed, our observation is in accordance with data coming from clinical samples of affective patients, suggesting that in IR patients, higher BMI is linked to lower anhedonia [64] and that increased levels of insulin might have a compensating role, normalizing responses to reward cues in overweight individuals with reduced insulin sensitivity [17,65].

Secondly, we observed that IR[+] vs. IR[−] participants were characterized by higher levels of anxious affective temperament and lower levels of hyperthymic affective temperament, conscientiousness, and emotional stability. What is more, several psychological variables were positive predictors of anhedonia: depressive, irritable, and anxious temperaments, cognitive disorganization, introvertive anhedonia, and overall schizotypy. Conversely, some personality traits were negative predictors of anhedonia: hyperthymic affective temperament, extraversion, and emotional stability. Similarly to Walsh et al., we noted that hyperthymic temperament is associated with higher positive affect (lower anhedonia), while depressive and anxious (analyzed together as a dysthymic temperament by Walsh et al., who used a different version of TEMPS-A) as well as irritable features are related to lower positive affect (higher anhedonia) [13]. Furthermore, our results are in congruence with those of Tobe et al. who reported that higher extraversion is linked to lower anhedonia [14]. Unlike in their work, we did not observe associations between agreeableness and openness to experience and anhedonia. This might be due to the use of a different tool for measuring the personality traits, and to the fact that our sample consisted mainly of patients with clinical diagnoses, while their sample included mainly healthy individuals. While our results suggested similar levels of schizotypy in IR[+] and IR[−] participants, Atbasoglu et al. reported that in a sample of unaffected siblings of schizophrenic and healthy individuals, higher schizotypy was linked to lower incidence of IR [66]. Again, the discrepancy might be due to differences in studied samples, as their analysis, unlike ours, included mainly HC, and also used a dissimilar tool to measure schizotypy. It might also be related to the fact that they used a different cut-off values of HOMA-IR indicating IR (≥2.7). Finally, our results showed that IR partially mediated associations between depressive affective temperament and anhedonia. 

This work should be seen in the context of its limitations. Anhedonia is a transdiagnostic phenomenon and we decided to assess it as such. Unfortunately, the majority of our sample consisted of MDD/FM patients, which makes it hard to compare our results with those previously reported. Other factors potentially confounding the analysis include the fact that we included individuals regardless of their nutritional status (normal, overweight, obese) and nicotine use, as well as our use of the TIPI inventory which, due to its brevity, does not have as high psychometric properties as longer tools constructed for the assessment of personality traits [52]. Next, the analysis was not planned a priori and in effect and the sample sizes were not calculated to detect the effects described in this particular analysis. Yet, given the novelty of our perspective merging the metabolic, symptomatic, and temperamental dimensions, no earlier data could inform such a priori calculations. Therefore, while our results should be considered exploratory, they constitute significant input to anhedonia research. Further studies need to investigate whether the relationships between state and trait anhedonia as well as IR are equally relevant in more homogenous groups of patients with clinical diagnoses or healthy subjects as well as subjects with normal BMI or overweight/obese individuals. Furthermore, our study is of cross-sectional design and therefore we cannot draw any conclusions on causal relationships based on it; hence, prospective studies assessing anhedonia as a multidimensional construct are much needed. Nevertheless, this work might be of high importance in the clinical management of subjects with MDD, FM, and other conditions characterized by marked anhedonia symptoms, as well as otherwise healthy subjects with marked anhedonia, as these individuals could benefit from interventions aimed at IR regardless of their constitutional temperamental, personality, or schizotypal traits. Moreover, psychological therapy could be helpful in anhedonic individuals, as it has been shown to improve emotional stability (reduce neuroticism) and extraversion (reduce introversion), as well as some domains of schizotypy [67,68]. Our results also provide an original multidimensional perspective on anhedonia and help disentangle the interrelationships between its many aspects, as well as setting the stage for future research.

## 5. Conclusions

The results indicate that IR, temperamental features, and personality traits are predictors of anhedonia levels. Further development of our comprehension of anhedonia and its state and trait aspects as well as its relation to insulin signaling and their interlinks are necessary to improve functional outcomes in not only MDD, but also in subjects with chronic pain, substance use disorders, schizophrenia, and Parkinson’s disease. A growing amount of data indicates the effectiveness of drugs which normalize insulin signaling on reward behavior and suggests a potential for repurposing of these drugs to target anhedonia [69]. More research is needed on insulin sensitizing therapies, including non-invasive lifestyle interventions, which could serve not only as add-ons for patients who experience anhedonia despite MDD treatment, but also for patients with addictions, chronic pain, and schizophrenia, or as a preventive strategy for healthy individuals who are prone to anhedonia.

## Figures and Tables

**Table 1:** Basic group characteristics.

Variable	IR[−] *n* = 69	IR[+] *n* = 69	IR[−] vs. IR[+] ^a,b^
Age mean years ± SD	43.33 ± 11.94	43.54 ± 12.44	t(136) = −0.978 *p* = 0.92 g = 0.02 negligible
Sex (female)	60	55	χ^2^(1138) = 0.835 *p* = 0.36
BMI kg/m^2^ ± mean	23.57 ± 3.81	28.32 ± 5.29	t(136) = −6.058 *p* < 0.001 g = 1.03 large
Hyperlipidemia (yes)	5	11	χ^2^(1138) = 1.767 *p* = 0.18
Hypertension (yes)	7	13	χ^2^(1138) = 1.462 *p* = 0.23
Hypothyroidism (yes)	12	6	χ^2^(1138) = 1.592 *p* = 0.1
Smoking (yes)	11	19	χ^2^(1138) = 2.087 *p* = 0.15
MDD/FM/HC	23/20/26	28/37/4	χ^2^(2138) = 21.694 *p* < 0.001

BMI, body mass index; FM, fibromyalgia; HC, healthy controls; IR[−], insulin sensitive subjects; IR[+], insulin resistant subjects; MDD, major depressive disorder; SD, standard deviation. ^a^ χ^2^ test was used to compare the qualitative data. A *t*-test was used to assess the differences in quantitative data. ^b^ g, Hedges g is the measure of effect size. Effect size lower than 0.2 was counted as negligible; 0.2–0.5 as small; 0.5–0.8 as medium; and >0.8 as large.

**Table 2:** Comparison of anhedonia, and features of temperament, personality, and schizotypal traits.

Variable	IR[−] Mean Score ± SD	IR[+] Mean Score ± SD	Test T IR[−] vs. IR[+] ^a^	p.corr
SHAPS	1.33 ± 2.05	2.74 ± 3.96	t(101.99) = −2.62 *p* = 0.01 g = 0.44 small	*p* = 0.033
TEMPS-A depressive	9.71 ± 3.8	11.48 ± 3.8	t(136) = −2.73 *p* = 0.007 g = 0.46 small	*p* = 0.052
TEMPS-A cyclothymic	7.10 ± 4.52	8.58 ± 5.08	t(136) = −1.81 *p* = 0.073 g = 0.31 small	*p* = 0.40
TEMPS-A hyperthymic	6.46 ± 3.58	5.35 ± 3.67	t(136) = 1.81 *p* = 0.07 g = 0.31 small	*p* = 0.012
TEMPS-A irritable	4.29 ± 3.39	5.48 ± 3.97	t(136) = −1.89 *p* = 0.06 g = 0.32 small	*p* = 0.37
TEMPS-A anxious	10.16 ± 5.58	13.58 ± 6.33	t(136) = −3.37 *p* < 0.001 g = 0.57 moderate	*p* = 0.02
TIPI extraversion	5.08 ± 1.58	4.51 ± 1.65	t(136) = 2.08 *p* = 0.04 g = 0.35 small	*p* = 0.051
TIPI agreeableness	5.46 ± 1.22	5.41 ± 1.33	t(136) = 0.23 *p* = 0.82 g = 0.04 negligible	*p* = 0.73
TIPI conscienciousness	5.62 ± 1.26	5.12 ± 1.68	t(126.08) = 2.00 *p* = 0.047 g = 0.34 small	*p* = 0.049
TIPI emotional stability	3.90 ± 1.6	3.12 ± 1.6	t(136) = 2.87 *p* = 0.005 g = 0.47 small	*p* = 0.005
TIPI openness to experience	4.40 ± 1.31	4.22 ± 1.28	t(136) = 0.78 *p* = 0.43 g = 0.13 negligible	*p* = 0.76
O-LIFE unusual experiences	5.64 ± 5.86	7.36 ± 6.09	t(136) = −1.69 *p* = 0.09 g = 0.29 small	*p* = 0.66
O-LIFE cognitive disorganization	10.72 ± 6.42	13.51 ± 6.65	t(136) = −2.50 *p* = 0.013 g = 0.42 small	*p* = 0.059
O-LIFE introvertive anhedonia	8.52 ± 4.88	10.30 ± 5.61	t(136) = −1.99 *p* = 0.049 g = 0.38 small	*p* = 0.37
O-LIFE impulsive nonconformity	6.1 3.82	7.3 4.39	t(136) = −1.72 *p* = 0.09 g = 0.29 small	*p* = 0.51
O-LIFE sum	30.99 ± 15.27	38.48 ± 16.92	t(136) = −2.73 *p* = 0.007 g = 0.46 small	*p* = 0.16

IR[−], insulin sensitive subjects; IR[+], insulin resistant subjects; p.corr, *p* value adjusted for BMI and hyperlipidemia (ANCOVA); SD, standard deviation; SHAPS, Snaith–Hamilton Pleasure Scale; O-LIFE, Oxford–Liverpool Inventory of Feelings and Experiences; TEMPS-A, Temperament Scale of Memphis, Pisa, and San Diego-autoquestionnaire; TIPI, Ten Item Personality Inventory; ^a^ g, Hedges g is the measure of effect size. Effect size lower than 0.2 was counted as negligible; 0.2–0.5 as small; 0.5–0.8 as medium; and >0.8 as large.

**Table 3:** Simple logistic regressions evaluating the associations between metabolic and psychological variables and the level of anhedonia.

Metabolic/Psychological Variable		Intercept	β	R^2^	*p*
BMI		1.784	0.01	−0.007	0.86
IR		1.227	0.323	0.026	0.032
TEMPS-A	depressive	−0.358	0.226	0.068	0.001
	cyclothymic	1.204	0.106	0.018	0.06
	hyperthymic	3.800	−0.299	0.109	<0.001
	irritable	1.227	0.166	0.030	0.024
	anxious	0.231	0.152	0.079	<0.001
TIPI	extraversion	6.570	−0.946	0.226	<0.001
	agreeableness	3.260	−0.225	0.001	0.29
	conscientiousness	4.435	−0.446	0.037	0.014
	emotional stability	4.238	−0.627	0.096	<0.001
	openness to experience	3.483	−0.336	0.011	0.11
O-LIFE	unusual experiences	2.112	−0.012	−0.007	0.80
	cognitive disorganization	0.09	0.161	0.104	<0.001
	introvertive anhedonia	−0.203	0.238	0.148	<0.001
	impulsive nonconfortmity	1.65	0.058	−0.002	0.39
	sum	0.196	0.053	0.067	0.001

BMI, body mass index; IR[−], insulin resistance; O-LIFE, Oxford–Liverpool Inventory of Feelings and Experiences; TEMPS-A, Temperament Scale of Memphis, Pisa, and San Diego-autoquestionnaire; TIPI, Ten Item Personality Inventory.

## Data Availability

The data presented in this study are available on request from the corresponding author due to privacy and ethics restrictions.

## Appendix A

**Table A1:** Mediation analyses evaluating the association between psychological variables (independent variable) and anhedonia (dependent variable) through metabolic variables.

TEMPS-A Depressive					
Through the HOMA-IR					
		Estimate CI	Lower 95% CI	Upper 95% CI	*p*
SHAPS	ACME	0.031263	0.000479	0.08	0.046
	ADE	0.194699	0.079928	0.33	<0.001
	Total effect	0.225962	0.109066	0.35	<0.001
	ACME/total effect	0.138355	0.001528	0.38	0.046
TEMPS-A cyclothymic					
Through the HOMA-IR					
		Estimate CI	Lower 95% CI	Upper 95% CI	*p*
SHAPS	ACME	0.02026	−0.00273	0.06	0.092
	ADE	0.08593	−0.01640	0.18	0.098
	Total effect	0.10620	0.00532	0.19	0.038
	ACME/total effect	0.19081	−0.07848	1.11	0.114
TEMPS-A hyperthymic					
Through the HOMA-IR					
		Estimate CI	Lower 95% CI	Upper 95% CI	*p*
SHAPS	ACME	−0.02311	−0.06113	0.00	0.094
	ADE	−0.27558	−0.46283	−0.13	<0.001
	Total effect	−0.29869	−0.49903	−0.14	<0.001
	ACME/total effect	0.07737	−0.00929	0.20	0.094
TEMPS-A irritable					
Through the HOMA-IR					
		Estimate CI	Lower 95% CI	Upper 95% CI	*p*
SHAPS	ACME	0.026799	0.000174	0.08	0.048
	ADE	0.138931	0.034065	0.27	0.006
	Total effect	0.165730	0.067724	0.29	<0.001
	ACME/total effect	0.161702	0.000924	0.58	0.048
TEMPS-A anxious					
Through the HOMA-IR					
		Estimate CI	Lower 95% CI	Upper 95% CI	*p*
SHAPS	ACME	0.02158	−0.00281	0.06	0.092
	ADE	0.13048	0.05532	0.21	0.002
	Total effect	0.15206	0.08135	0.23	<0.001
	ACME/total effect	0.14195	−0.01834	0.43	0.092
TIPI extraversion					
Through the HOMA-IR					
		Estimate CI	Lower 95% CI	Upper 95% CI	*p*
SHAPS	ACME	−0.04787	−0.12639	0.00	0.082
	ADE	−0.89804	−1.30337	−0.55	<0.001
	Total effect	−0.94591	−1.37545	−0.58	<0.001
	ACME/total effect	0.05061	−0.00344	0.13	0.082
TIPI agreeableness					
Through the HOMA-IR					
		Estimate CI	Lower 95% CI	Upper 95% CI	*p*
SHAPS	ACME	−0.0110	−0.1159	0.09	0.84
	ADE	−0.2144	−0.6280	0.19	0.31
	Total effect	−0.2253	−0.6304	0.16	0.30
	ACME/total effect	0.0487	−1.7021	1.63	0.86
TIPI conscientiousness					
Through the HOMA-IR					
		Estimate CI	Lower 95% CI	Upper 95% CI	*p*
SHAPS	ACME	−0.0686	−0.1635	0.00	0.062
	ADE	−0.3782	−0.9283	0.08	0.116
	Total effect	−0.4468	−1.0581	0.01	0.060
	ACME/total effect	0.1536	−0.2685	1.09	0.114
TIPI emotional stability					
Through the HOMA-IR					
		Estimate CI	Lower 95% CI	Upper 95% CI	*p*
SHAPS	ACME	−0.07068	−0.16966	0.00	0.036
	ADE	−0.55696	−0.91490	−0.24	<0.001
	Total effect	−0.62764	−1.00756	−0.29	<0.001
	ACME/total effect	0.11262	0.00436	0.26	0.036
TIPI openness to experience					
Through the HOMA-IR					
		Estimate CI	Lower 95% CI	Upper 95% CI	*p*
SHAPS	ACME	−0.0354	−0.1464	0.06	0.442
	ADE	−0.3002	−0.5942	0.05	0.080
	Total effect	0.3356	−0.6176	−0.02	0.038
	ACME/total effect	0.1054	−0.3773	0.98	0.468
O-LIFE unusual experiences					
Through the HOMA-IR					
		Estimate CI	Lower 95% CI	Upper 95% CI	*p*
SHAPS	ACME	0.01745	−0.00348	0.05	0.11
	ADE	−0.02903	−0.11988	0.06	0.50
	Total effect	−0.01158	−0.09301	0.07	0.77
	ACME/total effect	−1.50650	−8.99887	4.58	0.81
O-LIFE cognitive disorganization					
Through the HOMA-IR					
		Estimate CI	Lower 95% CI	Upper 95% CI	*p*
SHAPS	ACME	0.015856	0.000898	0.04	0.032
	ADE	0.144782	0.081679	0.22	<0.001
	Total effect	0.160639	0.090994	0.24	<0.001
	ACME/total effect	0.098707	0.007183	0.23	0.032
O-LIFE introvertive anhedonia					
Through the HOMA-IR					
		Estimate CI	Lower 95% CI	Upper 95% CI	*p*
SHAPS	ACME	0.016050	−0.000375	0.05	0.062
	ADE	0.221828	0.143448	0.31	<0.001
	Total effect	0.237878	0.159352	0.33	<0.001
	ACME/total effect	0.067472	−0.002029	0.19	0.062
O-LIFE impulsive nonconformity					
Through the HOMA-IR					
		Estimate CI	Lower 95% CI	Upper 95% CI	*p*
SHAPS	ACME	0.02411	−0.00189	0.06	0.076
	ADE	0.03353	−0.07885	0.15	0.572
	Total effect	0.05763	−0.05055	0.17	0.322
	ACME/total effect	0.41825	−3.55911	4.36	0.374
O-LIFE sum					
Through the HOMA-IR					
		Estimate CI	Lower 95% CI	Upper 95% CI	*p*
SHAPS	ACME	0.007382	0.000665	0.02	0.028
	ADE	0.045599	0.022539	0.07	<0.001
	Total effect	0.052981	0.029739	0.08	<0.001
	ACME/total effect	0.139327	0.012994	0.35	0.028
TEMPS-A depressive					
Through the BMI					
		Estimate CI	Lower 95% CI	Upper 95% CI	*p*
SHAPS	ACME	−0.00513	−0.04157	0.02	0.73
	ADE	0.23423	0.11253	0.37	<0.001
	Total effect	0.22909	0.11683	0.35	<0.001
	ACME/total effect	−0.02241	−0.17977	0.09	0.73
TEMPS-A cyclothymic					
Through the BMI					
		Estimate CI	Lower 95% CI	Upper 95% CI	*p*
SHAPS	ACME	−0.00201	−0.03131	0.02	0.846
	ADE	0.11348	0.03405	0.20	0.004
	Total effect	0.11147	0.02868	0.20	0.008
	ACME/total effect	−0.01804	−0.49003	0.17	0.854
TEMPS-A hyperthymic					
Through the BMI					
		Estimate CI	Lower 95% CI	Upper 95% CI	*p*
SHAPS	ACME	0.00200	−0.01077	0.02	0.74
	ADE	−0.30092	−0.47592	−0.14	<0.001
	Total effect	−0.29893	−0.47799	−0.14	<0.001
	ACME/total effect	−0.00668	−0.09759	0.03	0.74
TEMPS-A irritable					
Through the BMI					
		Estimate CI	Lower 95% CI	Upper 95% CI	*p*
SHAPS	ACME	−0.00395	−0.04353	0.02	0.82
	ADE	0.17440	0.06718	0.30	<0.001
	Total effect	0.17046	0.07462	0.30	<0.001
	ACME/total effect	−0.02315	−0.30626	0.15	0.82
TEMPS-A anxious					
Through the BMI					
		Estimate CI	Lower 95% CI	Upper 95% CI	*p*
SHAPS	ACME	−0.00648	−0.03663	0.01	0.51
	ADE	0.15987	0.08895	0.24	<0.001
	Total effect	0.15338	0.07975	0.23	<0.001
	ACME/total effect	−0.04227	−0.26364	0.06	0.51
TIPI extraversion					
Through the BMI					
		Estimate CI	Lower 95% CI	Upper 95% CI	*p*
SHAPS	ACME	0.00210	−0.02100	0.05	0.73
	ADE	−0.95277	−1.41380	−0.59	<0.001
	Total effect	−0.95068	−1.39832	−0.59	<0.001
	ACME/total effect	−0.00221	−0.05373	0.03	0.73
TIPI agreeableness					
Through the BMI					
		Estimate CI	Lower 95% CI	Upper 95% CI	*p*
SHAPS	ACME	0.000695	−0.043927	0.04	0.97
	ADE	−0.239233	−0.662102	0.18	0.25
	Total effect	−0.238539	−0.645843	0.18	0.25
	ACME/total effect	−0.002912	−0.393294	0.53	0.99
TIPI conscientiousness					
Through the BMI					
		Estimate CI	Lower 95% CI	Upper 95% CI	*p*
SHAPS	ACME	−0.000759	−0.029879	0.03	0.960
	ADE	−0.450360	−1.036009	−0.01	0.046
	Total effect	−0.451119	−1.028451	−0.02	0.042
	ACME/total effect	0.001682	−0.106690	0.16	0.966
TIPI emotional stability					
Through the BMI					
		Estimate CI	Lower 95% CI	Upper 95% CI	*p*
SHAPS	ACME	0.000490	−0.026118	0.04	0.98
	ADE	−0.631493	−1.005199	−0.30	<0.001
	Total effect	−0.631003	−0.993015	−0.30	<0.001
	ACME/total effect	−0.000777	−0.053523	0.06	0.98
TIPI openness to experience					
Through the BMI					
		Estimate CI	Lower 95% CI	Upper 95% CI	*p*
SHAPS	ACME	−0.000984	−0.053376	0.06	0.916
	ADE	−0.331312	−0.628457	−0.03	0.036
	Total effect	−0.332296	−0.620096	−0.04	0.032
	ACME/total effect	0.002962	−0.226472	0.26	0.924
O-LIFE unusual experiences					
Through the BMI					
		Estimate CI	Lower 95% CI	Upper 95% CI	*p*
SHAPS	ACME	0.00299	−0.02142	0.02	0.77
	ADE	−0.01127	−0.09152	0.08	0.81
	Total effect	−0.00827	−0.09266	0.07	0.86
	ACME/total effect	−0.36168	−3.24856	2.34	0.88
O-LIFE cognitive disorganization					
Through the BMI					
		Estimate CI	Lower 95% CI	Upper 95% CI	*p*
SHAPS	ACME	−0.00225	−0.01754	0.01	0.77
	ADE	0.16419	0.08595	0.25	<0.001
	Total effect	0.16195	0.09032	0.24	<0.001
	ACME/total effect	−0.01389	−0.09844	0.07	0.77
O-LIFE introvertive anhedonia					
Through the BMI					
		Estimate CI	Lower 95% CI	Upper 95% CI	*p*
SHAPS	ACME	−0.0115	−0.0488	0.01	0.36
	ADE	0.2505	0.1589	0.36	<0.001
	Total effect	0.2390	0.1547	0.34	<0.001
	ACME/total effect	−0.0479	−0.2129	0.04	0.36
O-LIFE impulsive nonconformity					
Through the BMI					
		Estimate CI	Lower 95% CI	Upper 95% CI	*p*
SHAPS	ACME	0.000103	−0.034148	0.03	0.94
	ADE	0.057530	−0.052075	0.18	0.28
	Total effect	0.057633	−0.045757	0.17	0.28
	ACME/total effect	0.001791	−1.738244	1.86	0.98
O-LIFE sum					
Through the BMI					
		Estimate CI	Lower 95% CI	Upper 95% CI	*p*
SHAPS	ACME	−0.00328	−0.01768	0.00	0.47
	ADE	0.05709	0.03145	0.09	<0.001
	Total effect	0.05381	0.03152	0.08	<0.001
	ACME/total effect	−0.06089	−0.32843	0.10	0.47

ACME, average causal mediation effects; ADE, average direct effect; BMI, body mass index; CI, confidence interval; HOMA-IR, homeostasis model assessment of insulin resistance; SHAPS, Snaith–Hamilton Pleasure Scale; O-LIFE, Oxford–Liverpool Inventory of Feelings and Experiences; TEMPS-A, Temperament Scale of Memphis, Pisa, and San Diego-autoquestionnaire; TIPI, Ten Item Personality Inventory.

## References

[1] Siwek M. (2017). Anhedonia in Depressive Disorders. J. Psychiatry Clin. Psychol..

[2] American Psychiatric Association (2013). Diagnostic and Statistical Manual of Mental Disorders.

[3] Trøstheim M., Eikemo M., Meir R., Hansen I., Paul E., Kroll S.L., Garland E.L., Leknes S. (2020). Assessment of Anhedonia in Adults with and without Mental Illness: A Systematic Review and Meta-Analysis. JAMA Netw. Open.

[4] Bair M.J., Robinson R.L., Katon W., Kroenke K. (2003). Depression and Pain Comorbidity. A Literature Review. Arch. Intern. Med..

[5] Calarco C.A., Lobo M.K. (2021). Depression and Substance Use Disorders: Clinical Comorbidity and Shared Neurobiology.

[6] Buckley P.F., Miller B.J., Lehrer D.S., Castle D.J. (2009). Psychiatric Comorbidities and Schizophrenia. Schizophr. Bull..

[7] Marsh L. (2013). Depression and Parkinson’s Disease: Current Knowledge. Curr. Neurol. Neurosci. Rep..

[8] Garland E.L., Trøstheim M., Eikemo M., Ernst G., Leknes S. (2019). Anhedonia in Chronic Pain and Prescription Opioid Misuse. Psychol. Med..

[9] Krupa A.J., Chrobak A.A., Sołtys Z., Korkosz M., Nowakowski J., Dudek D., Siwek M. (2024). Psychopathological Symptoms in Fibromyalgia and Their Associations with Resistance to Pharmacotherapy with SNRI. Psychiatr. Pol..

[10] Meehl P.E. (1962). Schizotaxia, Schizotypy, Schizophrenia. Am. Psychol..

[11] Mason O., Claridge G. (2006). The Oxford-Liverpool Inventory of Feelings and Experiences (O-LIFE): Further Description and Extended Norms. Schizophr. Res..

[12] Akiskal H.S., Placidi G.F., Maremmani I., Signoretta S., Liguori A., Gervasi R., Mallya G., Puzantian V.R. (1998). TEMPS-I: Delineating the Most Discriminant Traits of the Cyclothymic, Depressive, Hyperthymic and Irritable Temperaments in a Nonpatient Population. J. Affect. Disord..

[13] Walsh M.A., Brown L.H., Barrantes-Vidal N., Kwapil T.R. (2013). The Expression of Affective Temperaments in Daily Life. J. Affect. Disord..

[14] Tobe R.H., Tu L., Keefe J.R., Breland M.M., Ely B.A., Sital M., Richard J.T., Tural U., Iosifescu D.V., Gabbay V. (2023). Personality Characteristics, Not Clinical Symptoms, Are Associated with Anhedonia in a Community Sample: A Preliminary Investigation. J. Psychiatr. Res..

[15] Krupa A.J., Dudek D., Siwek M. (2023). Consolidating Evidence on the Role of Insulin Resistance in Major Depressive Disorder. Curr. Opin. Psychiatry.

[16] Martin H., Bullich S., Martinat M., Chataigner M., Di Miceli M., Simon V., Clark S., Butler J., Schell M., Chopra S. (2022). Insulin Modulates Emotional Behavior through a Serotonin-Dependent Mechanism. Mol. Psychiatry.

[17] Gruber J., Hanssen R., Qubad M., Bouzouina A., Schack V., Sochor H., Schiweck C., Aichholzer M., Matura S., Slattery D.A. (2023). Impact of Insulin and Insulin Resistance on Brain Dopamine Signalling and Reward Processing—An Underexplored Mechanism in the Pathophysiology of Depression?. Neurosci. Biobehav. Rev..

[18] Singh M.K., Leslie S.M., Packer M.M., Zaiko Y.V., Phillips O.R., Weisman E.F., Wall D.M., Jo B., Rasgon N. (2019). Brain and Behavioral Correlates of Insulin Resistance in Youth with Depression and Obesity. Horm. Behav..

[19] Brouwer A., van Raalte D.H., Lamers F., Rutters F., Elders P.J.M., Van Someren E.J.W., Snoek F.J., Beekman A.T.F., Bremmer M.A. (2021). Insulin Resistance as a Marker for the Immune-Metabolic Subtype of Depression. J. Affect. Disord..

[20] Rashidian H., Subramaniapillai M., Park C., Lipsitz O., Zuckerman H., Teopiz K., Cao B., Lee Y., Gill H., Ho R. (2021). Insulin Resistance Is Associated with Deficits in Hedonic, Self-Reported Cognitive, and Psychosocial Functional Response to Antidepressant Treatment in Individuals with Major Depressive Disorder. J. Affect. Disord..

[21] Rashidian H., Subramaniapillai M., Park C., Lipsitz O., Zuckerman H., Cao B., Lee Y., Gill H., Rodrigues R.N., Di Vincenzo J.D. (2023). Changes in Insulin Resistance Following Antidepressant Treatment Mediate Response in Major Depressive Disorder. J. Psychopharmacol..

[22] Krupa A.J., Chrobak A.A., Sołtys Z., Dudek D., Szewczyk B., Siwek M. (2024). Insulin Resistance, Clinical Presentation and Resistance to Selective Serotonin and Noradrenaline Reuptake Inhibitors in Major Depressive Disorder. Pharmacol. Rep..

[23] Nunnari P., Ceccarelli G., Ladiana N., Notaro P. (2021). Prescribing Cascades and Medications Most Frequently Involved in Pain Therapy: A Review. Eur. Rev. Med. Pharmacol. Sci..

[24] Marasine N.R., Sankhi S., Lamichhane R., Marasini N.R., Dangi N.B. (2021). Use of Antidepressants among Patients Diagnosed with Depression: A Scoping Review. BioMed Res. Int..

[25] Fagiolini A., Florea I., Loft H., Christensen M.C. (2021). Effectiveness of Vortioxetine on Emotional Blunting in Patients with Major Depressive Disorder with Inadequate Response to SSRI/SNRI Treatment. J. Affect. Disord..

[26] Price J., Cole V., Goodwin G.M. (2009). Emotional Side-Effects of Selective Serotonin Reuptake Inhibitors: Qualitative Study. Br. J. Psychiatry.

[27] Goodwin G.M., Price J., De Bodinat C., Laredo J. (2017). Emotional Blunting with Antidepressant Treatments: A Survey among Depressed Patients. J. Affect. Disord..

[28] Cipriani A., Furukawa T.A., Salanti G., Chaimani A., Atkinson L.Z., Ogawa Y., Leucht S., Ruhe H.G., Turner E.H., Higgins J.P.T. (2018). Comparative Efficacy and Acceptability of 21 Antidepressant Drugs for the Acute Treatment of Adults with Major Depressive Disorder: A Systematic Review and Network Meta-Analysis. Lancet.

[29] Qaseem A., Owens D.K., Etxeandia-Ikobaltzeta I., Tufte J., Cross J.T., Wilt T.J., Carroll K., Shamliyan T., Yost J. (2023). Nonpharmacologic and Pharmacologic Treatments of Adults in the Acute Phase of Major Depressive Disorder: A Living Clinical Guideline from the American College of Physicians. Ann. Intern. Med..

[30] Krupa A.J., Wojtasik-Bakalarz K., Siwek M. (2023). Vortioxetine—Pharmacological Properties and Use in Mood Disorders. The Current State of Knowledge. Psychiatr. Pol..

[31] Cuomo A., Ballerini A., Bruni A.C., Decina P., Di Sciascio G., Fiorentini A., Scaglione F., Vampini C., Fagiolini A. (2019). Clinical Guidance for the Use of Trazodone in Major Depressive Disorder and Concomitant Conditions: Pharmacology and Clinical Practice. Riv. Psichiatr..

[32] Stahl S.M. (2015). Modes and Nodes Explain the Mechanism of Action of Vortioxetine, a Multimodal Agent (MMA): Enhancing Serotonin Release by Combining Serotonin (5HT) Transporter Inhibition with Actions at 5HT Receptors (5HT1A, 5HT1B, 5HT1D, 5HT7 Receptors). CNS Spectr..

[33] Dudek D., Chrobak A.A., Krupa A.J., Gorostowicz A., Gerlich A., Juryk A., Siwek M. (2023). TED—Trazodone Effectiveness in Depression: A Naturalistic Study of the Effeciveness of Trazodone in Extended Release Formulation Compared to SSRIs in Patients with a Major Depressive Disorder. Front. Pharmacol..

[34] Siwek M., Gorostowicz A., Chrobak A.A., Gerlich A., Krupa A.J., Juryk A., Dudek D. (2023). TED—Trazodone Efficacy in Depression: A Naturalistic Study on the Efficacy of Trazodone in an Extended-Release Formulation Compared to SSRIs in Patients with a Depressive Episode—Preliminary Report. Brain Sci..

[35] Siwek M., Chrobak A.A., Krupa A.J., Gorostowicz A., Gerlich A., Juryk A., Dudek D. (2023). TED (Trazodone Effectiveness in Depression): Effectiveness of Trazodone Extended-Release in Subjects with Unsatisfactory Response to SSRIs. Psychiatr. Pol..

[36] Corruble E., De Bodinat C., Belaïdi C., Goodwin G.M., Burrows G., Lopes Rocha F., Bakish D., Emsley R., Avedisova A., Hale A. (2013). Efficacy of Agomelatine and Escitalopram on Depression, Subjective Sleep and Emotional Experiences in Patients with Major Depressive Disorder: A 24-Wk Randomized, Controlled, Double-Blind Trial. Int. J. Neuropsychopharmacol..

[37] De Berardis D., Fornaro M., Orsolini L., Iasevoli F., Tomasetti C., De Bartolomeis A., Serroni N., De Lauretis I., Girinelli G., Mazza M. (2017). Effect of Agomelatine Treatment on C-Reactive Protein Levels in Patients with Major Depressive Disorder: An Exploratory Study in Real-World, Everyday Clinical Practice. CNS Spectr..

[38] Pochwat B., Krupa A.J., Siwek M., Szewczyk B. (2022). New Investigational Agents for the Treatment of Major Depressive Disorder. Expert Opin. Investig. Drugs.

[39] Nogo D., Jasrai A.K., Kim H., Nasri F., Ceban F., Lui L.M.W., Rosenblat J.D., Vinberg M., Ho R., McIntyre R.S. (2022). The Effect of Ketamine on Anhedonia: Improvements in Dimensions of Anticipatory, Consummatory, and Motivation-Related Reward Deficits. Psychopharmacology.

[40] Wolfe F., Clauw D.J., Fitzcharles M.A., Goldenberg D.L., Häuser W., Katz R.L., Mease P.J., Russell A.S., Russell I.J., Walitt B. (2016). 2016 Revisions to the 2010/2011 Fibromyalgia Diagnostic Criteria. Semin. Arthritis Rheum..

[41] Tyrer P., Reed G.M., Crawford M.J. (2015). Classification, Assessment, Prevalence, and Effect of Personality Disorder. Lancet.

[42] Kleykamp B.A., Ferguson M.C., McNicol E., Bixho I., Arnold L.M., Edwards R.R., Fillingim R., Grol-Prokopczyk H., Turk D.C., Dworkin R.H. (2021). The Prevalence of Psychiatric and Chronic Pain Comorbidities in Fibromyalgia: An Acttion Systematic Review. Semin. Arthritis Rheum..

[43] Matthews D.R., Hosker J.P., Rudenski A.S., Naylor B.A., Treacher D.F., Turner R.C. (1985). Homeostasis Model Assessment: Insulin Resistance and β-Cell Function from Fasting Plasma Glucose and Insulin Concentrations in Man. Diabetologia.

[44] Wallace T.M., Levy J.C., Matthews D.R. (2004). Use and Abuse of HOMA Modeling. Diabetes Care.

[45] Szurkowska M., Krystyna S., Gilis-Januszewska A., Szybiński Z., Huszno B. (2005). Insulin Resistance Indices in Population-Based Study and Their Predictive Value in Defining Metabolic Syndrome. Epidemiol. Rev..

[46] Placzkowska S., Pawlik-Sobecka L., Kokot I., Piwowar A. (2019). Indirect Insulin Resistance Detection: Current Clinical Trends and Laboratory Limitations. Biomed. Pap..

[47] Snaith R.P., Hamilton M., Morley S., Humayan A., Hargreaves D., Trigwell P. (1995). A Scale for the Assessment of Hedonic Tone. The Snaith-Hamilton Pleasure Scale. Br. J. Psychiatry.

[48] Franken I.H.A., Rassin E., Muris P. (2007). The Assessment of Anhedonia in Clinical and Non-Clinical Populations: Further Validation of the Snaith-Hamilton Pleasure Scale (SHAPS). J. Affect. Disord..

[49] Gorostowicz A., Rizvi S.J., Kennedy S.H., Chrobak A.A., Dudek D., Cyranka K., Piekarska J., Krawczyk E., Siwek M. (2023). Polish Adaptation of the Dimensional Anhedonia Rating Scale (DARS)—Validation in the Clinical Sample. Front. Psychiatry.

[50] Dembińska-Krajewska D., Rybakowski J. (2017). The Temperament Evaluation of Memphis, Pisa and San Diego Autoquestionnaire (TEMPS-A)—An Important Tool to Study Affective Temperaments the Temperament Evaluation of Memphis, Pisa and San Diego Auto-Questionnaire (TEMPS-A)—An Important Tool to Study Affective Temperaments. Psychiatr. Pol..

[51] Borkowska A., Rybakowski J.K., Drozdz W., Bielinski M., Kosmowska M., Rajewska-Rager A., Bucinski A., Akiskal K.K., Akiskal H.S. (2010). Polish Validation of the TEMPS-A: The Profile of Affective Temperaments in a College Student Population. J. Affect. Disord..

[52] Sorokowska A., Słowińska A., Zbieg A., Sorokowski P. (2013). Polska Adaptacja Testu TIPI.

[53] Thørrisen M.M., Sadeghi T. (2023). The Ten-Item Personality Inventory (TIPI): A Scoping Review of Versions, Translations and Psychometric Properties. Front. Psychol..

[54] Dembińska-Krajewska D., Rybakowski J. (2014). The Oxford-Liverpool Inventory of Feelings and Experiences (O-LIFE) Schizotypy Scale in Psychiatry. Arch. Psychiatry Psychother..

[55] Alminhana L.O., Sanseverino M.A., Farias M., Dos Santos O.V., De Lara Machado W., Claridge G. (2020). A Dimensional Measure of Schizotypy: Cross-Cultural Adaptation and Validation of the Oxford-Liverpool Inventory of Feelings and Experiences Short Version for Brazilian Portuguese (o-Life-S). Trends Psychiatry Psychother..

[56] Krupa A.J., Korkosz M., Gorostowicz A., Nowakowski J., Kierzkowska I., Dudek D., Siwek M. (2023). Predictors of Treatment Response to Serotonin and Noradrenaline Reuptake Inhibitors in Fibromyalgia. Pol. Arch. Intern. Med..

[57] Krupa A.J., Chrobak A.A., Sołtys Z., Korkosz M., Dudek D., Siwek M. (2023). Psychological Variables Associated with Resistance to Treatment with Serotonin and Noradrenaline Reuptake Inhibitors in Fibromyalgia. J. Psychosom. Res..

[58] Krupa A.J., Chrobak A.A., Sołtys Z., Korkosz M., Nowakowski J., Dudek D., Siwek M. (2024). Chronobiological Variables Predict Non-Response to Serotonin and Noradrenaline Reuptake Inhibitors in Fibromyalgia: A Cross-Sectional Study. Rheumatol. Int..

[59] R CoreTeam (2022). A Language and Environment for Statistical Computing.

[60] Tingley D., Yamamoto T., Hirose K., Keele L., Imai K. (2015). Healthy Longevity in China: Demographic, Socioeconomic, and Psychological Dimensions. J. Stat. Softw..

[61] Wickham H. (2016). ggplot2: Elegant Graphics for Data Analysis.

[62] Mansur R.B., Subramaniapillai M., Zuckerman H., Park C., Iacobucci M., Lee Y., Tuineag M., Hawco C., Frey B.N., Rasgon N. (2019). Effort-Based Decision-Making Is Affected by Overweight/Obesity in Major Depressive Disorder. J. Affect. Disord..

[63] Cho J., Goldenson N.I., Pester M.S., Bello M.S., Dunton G.F., Britni R., Leventhal A.M., Angeles L., Angeles L. (2018). Longitudinal Associations between Anhedonia and BMI Trajectory Groups among Adolescents Junhan. J. Adolesc. Health.

[64] Mansur R.B., Brietzke E., Lee Y., Subramaniapillai M., Cha D.S., McIntyre R.S. (2019). Parsing Metabolic Heterogeneity in Mood Disorders: A Hypothesis—Driven Cluster Analysis of Glucose and Insulin Abnormalities. Bipolar Disord..

[65] Schneider E., Spetter M.S., Martin E., Sapey E., Yip K.P., Manolopoulos K.N., Tahrani A.A., Thomas J.M., Lee M., Hallschmid M. (2022). The Effect of Intranasal Insulin on Appetite and Mood in Women with and without Obesity: An Experimental Medicine Study. Int. J. Obes..

[66] Atbasoglu E.C., Gumus-Akay G., Guloksuz S., Saka M.C., Ucok A., Alptekin K., Gullu S., van Os J. (2018). Higher Schizotypy Predicts Better Metabolic Profile in Unaffected Siblings of Patients with Schizophrenia. Psychopharmacology.

[67] Hengartner M.P., von Wyl A., Heiniger Haldimann B., Yamanaka-Altenstein M. (2020). Personality Traits and Psychopathology Over the Course of Six Months of Outpatient Psychotherapy: A Prospective Observational Study. Front. Psychol..

[68] Grant P., Munk A.J.L., Hennig J. (2018). A Positive-Psychological Intervention Reduces Acute Psychosis-Proneness. Schizophr. Res..

[69] Badulescu S., Tabassum A., Le G.H., Wong S., Phan L., Gill H., Llach C.D., McIntyre R.S., Rosenblat J., Mansur R. (2024). Glucagon-like Peptide 1 Agonist and Effects on Reward Behaviour: A Systematic Review. Physiol. Behav..

